# Non-Invasive Methods for the Secondary and Tertiary Prevention of Early Childhood Caries: A Scoping Review

**DOI:** 10.3390/healthcare14010064

**Published:** 2025-12-26

**Authors:** Agnieszka Wasiluk, Katarzyna Domosławska-Żylińska, Dominik Olejniczak

**Affiliations:** 1 Department of Health Promotion and Prevention of Chronic Diseases, National Institute of Public Health NIH—National Research Institute, 24 Chocimska St, 00-791 Warsaw, Poland; 2 Department of Public Health, Medical University of Warsaw, 61 Żwirki i Wigury St., 02-091 Warsaw, Poland

**Keywords:** early childhood caries, primary teeth, silver diamine fluoride, sodium fluoride

## Abstract

**Background:** Early childhood caries is defined as a carious disease affecting primary teeth in children under 6 years of age. It may lead to pain, infections, and difficulties with eating. Despite its burden, evidence on simple, non-invasive preventive approaches which can be implemented both in dental clinics and outreach services is fragmented. The aim of this review was to identify and map such methods for the secondary and tertiary prevention of ECC and to define priorities for future research. **Material and Methods:** The scoping review followed the PCC framework (Population–Concept–Context). Two databases were searched: PubMed and Scopus. A systematic search was conducted in PubMed and Scopus between 1 August and 30 September 2025. Eligible studies included children under 6 years of age with existing carious lesions, evaluated non-invasive methods for secondary and tertiary ECC prevention (such as sodium fluoride (NaF), silver diamine fluoride (SDF), nano-silver fluoride (NSF), and motivational techniques), requiring simple armamentarium, and reported data on the effectiveness in the context of ECC. Only publications from the past 5 years, available in English, and in open access, were considered. The results of the analysis were summarized narratively, outlining intervention types based on their characteristics, impact, and usage context. **Results:** Fifteen studies were included. Most were randomized controlled trials (eight studies), focusing primarily on silver diamine fluoride (SDF), often compared with other non-invasive methods, followed by systematic reviews (two studies), reviews (two studies), cross-sectional studies (two articles), and one qualitative study. Only one publication examined the use of motivational interviewing within the context of ECC. While the evidence on non-invasive approaches is growing, significant gaps remain. Small sample sizes, short follow-up periods, and heterogenous interventions and outcomes limit comparability. To strengthen the evidence base, future studies should recruit larger cohorts, adhere to standardized procedures, and use consistent reporting. **Conclusions:** The majority of studies focused on SDF, reflecting the increasing interest in its use. Research on motivational interviewing in ECC is particularly scarce. Further research under standardized conditions is needed to enable reliable comparisons across treatment protocols.

## 1. Introduction

Dental caries is the most common disease of the masticatory system. It arises through the activity of dental biofilm and results in the progressive destruction of the tooth’s hard tissues. The core mechanism of the disease is the demineralization of these tissues due to a drop in pH caused by acids produced by bacteria on the tooth surface during carbohydrate fermentation (mainly lactic acid). This is followed by the disintegration of the organic substances contained within the tooth structure [1].

Early childhood caries (ECC) is defined as a carious disease affecting primary teeth in children under the age of six. It is characterized by the presence of one or more decayed, missing, or filled (due to caries) teeth in children aged 5 years and 11 months or younger (children younger than 6 years old) [1]. This issue represents a serious public health concern globally.

The World Health Organization (WHO) identifies children aged 5 as a key age group for assessing the prevalence of dental caries in primary teeth within the population. At this stage, the severity and consequences of early childhood caries (ECC) can be fully evaluated. Among European countries, Poland reports the highest prevalence of ECC in 5-year-olds (76.7% in 2016), whereas Germany shows the lowest (26.2% in 2014) [2]. The highest increase in the incidence of caries in primary teeth occurs during the first 3 years of life, followed by a further rise over the next 2 years, during which the prevalence of caries increases by 35.6% [3].

ECC may lead to a range of health complications, including pain, infections, reduced appetite, and difficulties with eating—all of which can adversely affect a child’s growth, development, and overall well-being [4]. Primary prevention of dental caries aims to prevent the onset of the disease (maintaining the balance between the remineralization and demineralization) through measures such as proper oral hygiene, healthy diet, fluoride treatment, and regular check-ups. Secondary prevention focuses on the early detection of carious lesions and halting their progression, whereas tertiary caries prevention aims at managing the consequences of advanced dental caries and incorporates measures from both primary and secondary prevention [5].

This article discusses selected methods for the secondary and tertiary prevention of non-invasive treatment for early childhood caries. According to ICDAS (International Caries Detection and Assessment System) detection codes for coronal caries (where score 0 means a healthy enamel, scores 1–2 initial carious lesions, scores 3–4 moderate lesions, and scores 5–6 the advanced carious lesions) [1], ICDAS 1–2 means that lesions are still reversible and can be arrested without surgery intervention, and that there is no loss of enamel integrity (secondary prevention, e.g., fluoride application, dietary changes). ICDAS 3 and above means that the loss of structural integrity of the enamel or/and dentin has occurred, what requires more advanced treatment (these actions are considered as tertiary prevention).

This article will devote considerable attention to SDF (silver diamine fluoride—although it is often used for moderate and advanced carious lesions) and its innovative modifications, as it fits within the model of non-invasive treatment of early childhood caries.

The treatment of dental caries may follow either a medical (non-operative or non-invasive) or a surgical (operative or invasive) approach [6]. In recent years, there has been growing emphasis on minimally invasive and non-invasive approaches, particularly in pediatric populations, to reduce treatment-related anxiety and promote positive dental experiences. Primary prevention of early childhood caries (ECC) is effective, but if caries does occur and is detected, especially at an early stage, the available therapeutic non-restorative methods within secondary or tertiary prevention can be successfully applied. The earlier the carious lesion is identified, the greater the chance of halting its progression.

Non-restorative treatment refers to a preventive and therapeutic approach that avoids the placement of traditional restorations (which periodically require repair or replacement). It encompasses a range of minimally invasive interventions such as silver diamine fluoride (SDF) application, silver nitrate impregnation, resin infiltration (e.g., ICON), sodium fluoride (NaF) and other remineralizing agents, ozone therapy, as well as behavioral strategies including oral hygiene instruction, dietary counseling, and biofilm management. In the treatment of early childhood caries, the most desirable methods are those which are simple, quick, cost-effective, do not involve complicated armamentarium and high qualified personnel, and can be conducted also in other places than dental clinics.

Children are truly exceptional patients—open-hearted, trusting, and often vulnerable when faced with unfamiliar situations. Their sensitivity and honesty make every dental visit an opportunity not only for treatment, but also for building lifelong attitudes toward oral health. Positive dental experiences are crucial for children, regardless of the stage of carious disease. Evidence-based dentistry provides a broad spectrum of interventions tailored to clinical needs, ranging from restorative procedures to preventive and non-invasive approaches. While non-invasive methods may not always achieve the same outcomes as restorative treatment—particularly in advanced lesions—they play an important role in managing early childhood caries in a way that is simple, swift, painless, and developmentally appropriate. These techniques provide satisfactory clinical results while fostering trust and cooperation, making treatment both effective and gentle [7].

This article presents the most commonly used contemporary methods that meet the outlined criteria, including the application of 5% sodium fluoride (NaF), 38% silver diamine fluoride (SDF) in various intervals and combinations, as well as SDF modern nanotechnology-based modifications designed to mitigate its most significant side effect (a black discoloration of carious lesions).

Although various non-invasive approaches have been proposed for managing ECC, the existing literature lacks a clear overview of these interventions. There is limited clarity regarding their scope, clinical application, and reported outcomes.

The aim of this scoping review was to identify and map non-invasive methods used in the secondary and tertiary prevention of early childhood caries (ECC) that are simple, require minimal equipment and personnel, and can be implemented both in dental clinics and outreach services. Additionally, the review sought to define the scope of the existing evidence and highlight directions for future research in this area. The study was designed as a scoping review in accordance with the methodological guidelines of the Joanna Briggs Institute and reported following the PRISMA-ScR recommendations. Consistent with the purpose of scoping reviews, the study was conducted within predefined scope limitations, including restricted database selection, language filters, and a defined time frame.

## 2. Materials and Methods

The review process was based on the PCC framework (Population–Concept–Context), which enables the appropriate formulation of the scope of scoping review. The population consisted of children under 6 years of age with existing carious lesions. The concept included non-invasive methods for secondary and/or tertiary prevention that can be performed in a simple and quick manner, included outreach service (e.g., in a preschool), such as the application of fluoride varnish (NaF), silver diamine fluoride (SDF), nano-silver fluoride (NSF), and motivational techniques. The context of the review was not geographically restricted and encompassed various healthcare settings and socioeconomic conditions.

A systematic literature search was conducted between 1 August and 30 September 2025. Two databases were searched: PubMed and Scopus. The “last search” date for PubMed was 30 September 2025 and the “last search” date for Scopus was also 30 September 2025. The search strategy was developed based on a combination of keywords and MeSH terms. Search strings in PubMed were as follows: “Early Childhood Caries” OR “ECC” AND “nonrestorative” OR “nonrestorative treatment” AND “primary teeth” OR “milk teeth” OR “primary dentition” AND “fluoride varnish” OR “silver nitrate” OR “silver diamine fluoride” OR “resin infiltration” OR “ICON”. Additional filters were applied: last 5 years (publication year), free full text (text availability), clinical trial, meta-analysis, randomized clinical trial, review, systematic review (article type), English (article language), and Child: birth-18 years (age). The filter Child: birth-18 years was applied to broadly narrow the search scope. Subsequently, studies were screened in a stepwise manner: first by title and abstract, and then through full-text assessment. In line with the eligibility criteria and the standard age definition of early childhood caries, only studies involving children under 6 years of age were included, while studies with participants aged 6 years and older were excluded. Search strings in Scopus were as follows: “Early Childhood Caries” OR “ECC” AND “nonrestorative” OR “nonrestorative treatment” AND “primary teeth” OR “milk teeth” OR “primary dentition” AND “fluoride varnish” OR “silver nitrate” OR “silver diamine fluoride” OR “resin infiltration” OR “ICON”. Additional filters were applied: 2021–2025 (publication year), open access (access type), Dentistry, Medicine (subject area), article, review (document type), English (language). The review was not registered in PROSPERO or OSF, which constitutes one of the methodological limitations of the study.

The review included original empirical studies, including randomized controlled trials (RCTs), cohort studies, and cross-sectional studies. Selected review papers (systematic and narrative) were also considered for supportive purposes. Studies were eligible for analysis if the population consisted of children under 6 years of age with symptoms of caries, and the interventions were exclusively non-invasive—such as the use of silver diamine fluoride (SDF), nano-silver fluoride (NSF), sodium fluoride (NaF), health education, dietary changes, or motivational interviewing. Studies involving restorative interventions (e.g., fillings or crowns) and advanced equipment (such as laser or ozone generators), populations older than 5 years, and publications lacking data on the effectiveness of the applied methods were excluded from the review. The selection of review papers was limited to those published in the past five years, addressing non-invasive approaches (only those that are quick and simple) to ECC, written in English, and available as full-text open access.

The inclusion and exclusion criteria are summarized in Table 1.

As part of the search strategy, reference lists of included studies were screened selectively to identify additional relevant publications. However, no further articles met the inclusion criteria. No other supplementary methods were employed.

The selection of publications was conducted in two stages. In the first stage, titles and abstracts were reviewed, while in the second stage, full texts of the eligible studies were assessed. The selection process was carried out by two independent authors (AW and KDŻ), and any discrepancies were resolved through discussion or consultation with a third author (DO). Data from the included articles were extracted into a standardized data sheet which included bibliographic information (author, title, year of publication, country), study type, characteristics of the study population, type of intervention, follow-up periods, key results, and the authors’ main conclusions. Details are provided in the Supplementary Materials.

The results were presented as a narrative descriptive synthesis, including a mapping of intervention types according to their nature, effectiveness, and context of application. In accordance with PRISMAScR and JBI guidance, no formal quality or risk of bias assessment was conducted, as the primary aim of this review was to map the existing literature rather than evaluate the strength of evidence or perform a restrictive analysis.

This scoping review has several limitations: the absence of a registered a priori protocol, which may reduce transparency and increase susceptibility to selection or outcome reporting bias due to the lack of a publicly available methodological framework; the potential omission of unpublished studies (gray literature); language restrictions (English-only publications); and the inconsistent definitions of “non-restorative” or “non-invasive” or “minimally invasive” methods of secondary and tertiary prevention in the available literature, which may have influenced study selection. No formal risk of bias assessment was conducted, in line with scoping review methodology (PRISMA-ScR, JBI), though this limits the interpretation of evidence strength. This review was based solely on PubMed and Scopus, and the omission of other databases may have further narrowed the scope.

## 3. Results

A total of 15 studies met the eligibility criteria and were included in the final synthesis. The included studies were published within the last five years, with the majority originating from China (five articles) and Egypt (four articles). The body of literature consisted primarily of randomized controlled trials (eight studies), followed by systematic reviews (two studies), reviews (two studies), cross-sectional studies (two articles), and one qualitative study.

**Figure 1:**
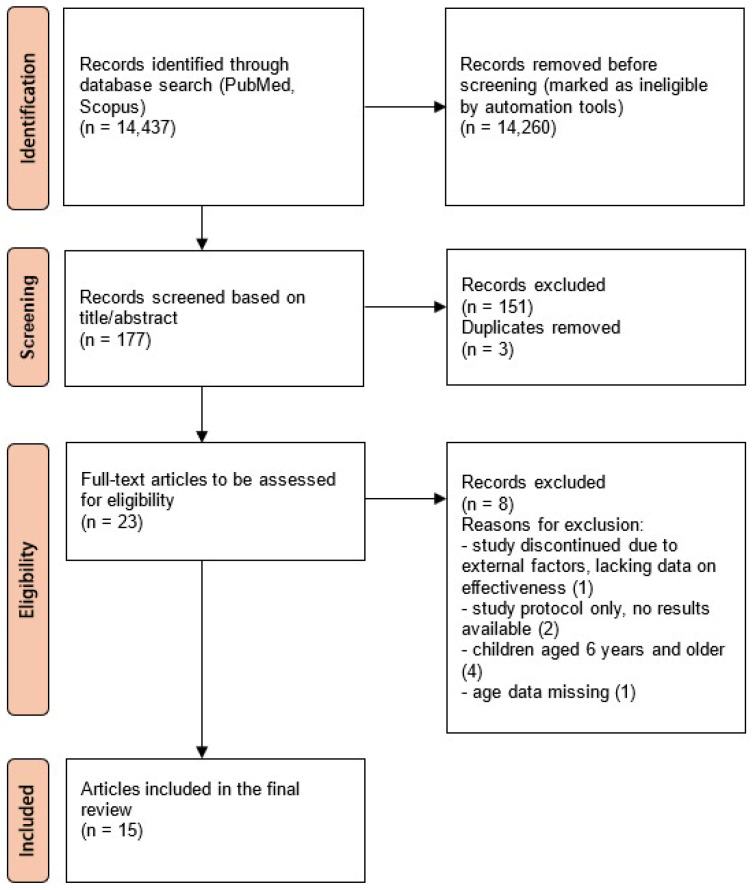
PRISMA flow diagram. The numbers in parentheses next to the reasons for exclusion indicate the number of articles excluded for each reason.

The study selection process was illustrated using a PRISMA flow diagram (Figure 1).

The findings were grouped into three main themes:

1. Characteristics, safety profile, innovations, and adverse effects of silver diamine fluoride (SDF)—two articles.
2. Use of silver and fluoride compounds (primarily 38% SDF and 5% NaF) in the treatment of early childhood caries at various intervals and in different combinations—nine articles.
3. Perceptions of SDF among parents and children, as well as the use of parental motivational interviewing (MI) in pediatric dentistry—four articles.

### 3.1. Characteristics, Safety Profile, Innovations, and Adverse Effects of SDF

The first article was a review study and it mainly referred to the characteristics, safety, and adverse effects of SDF. Developed in Japan in 1960, silver diamine fluoride (SDF) is commonly used as a 38% alkaline, colorless solution containing silver, fluoride, and ammonia (added to stabilize the compound as silver diamine fluoride). SDF is photosensitive and decomposes upon light exposure, releasing silver, which has antibacterial properties. Fluoride supports remineralization, and together they offer a synergistic effect in arresting caries. SDF is considered to be safe, with the main drawback being the permanent black staining of carious lesions. It may also cause a metallic taste and temporary irritation of soft tissues, which resolve without treatment. Its simplicity allows application not only by dentists but also physicians and trained health personnel. The authors also emphasize that SDF is non-aerosol-generating, what reduces the risk of cross-infection [8].

The second publication is also a review article and it concentrates on the advancements in SDF-based caries management. The authors emphasize the importance of procedural simplicity in the application of silver diamine fluoride (SDF), which makes it accessible to a wide range of healthcare providers. However, the most notable adverse effect remains the irreversible black staining of carious lesions. This discoloration results from the oxidation of ionic silver into metallic silver and silver dioxide, followed by the precipitation of silver–protein and silver–phosphate complexes on the tooth surface. The color change begins within two minutes of application and reaches its peak after approximately 12 h. To reduce staining, researchers are investigating nanomaterial-based antimicrobial agents such as silver, selenium, and copper nanoparticles. Their properties can be tailored to enhance efficacy. Nano-silver fluoride (NSF) is considered a promising alternative, offering similar antibacterial effects as SDF but without discoloration [7].

### 3.2. Silver Diamine Fluoride (SDF) and Sodium Fluoride (NaF) in the Management of Early Childhood Caries (ECC)

These compounds were used in various studies in a wide range of combinations. Eight articles with randomized controlled trials and one review article were found on this topic.

Two clinical studies compared the effectiveness of 38% SDF with 5% NaF in caries arrestment in primary teeth [9,10]. In one of them, agents were applied only on the upper anterior teeth, and 12-month observation found that a single application of 38% SDF was not superior to 5% NaF for caries prevention [10]. In the second study, researchers also concluded that there was no significant difference in arresting enamel caries among these agents (in this study, agents were applied semiannually—final control visit was after 18 months) [9].

In another study, 38% SDF was compared with nano-silver fluoride (NSF) and it was concluded that the caries arrest rate was significantly higher in the NSF than SDF group (78.4% and 65% at 6 months, respectively, and 71.3% and 56.3% at 12 months, respectively, *p* < 0.001) [11].

There was also a publication in which authors compared single application of 38% SDF with 5% NaF followed by motivational interviewing (MI) sessions (one at baseline and the second after three months, only in the NaF group). It concluded that 5% NaF/MI can be an alternative to 38% SDF in arresting advanced and moderate carious lesions without staining, with a stronger effect on moderate lesions [12].

Another study addressed the comparison between the semiannual application of 38% SDF and semiannual application of 25% silver nitrate along with 5% NaF—it concluded that 25% silver nitrate with 5% NaF is at least as effective as the application of 38% SDF in arresting early childhood caries, if applied semiannually [13].

In one publication, three different methods were compared. The study compared 38% SDF with atraumatic restorative treatment (ART) and with ultraconservative treatment (UCT), but it was conducted only on the occlusal surfaces of primary molars. The final remarks of this study indicated that the caries arrest rate of UCT, after 1 year of observation, was significantly lower than SDF and ART (the overall success rates of UCT, SDF, and ART were 61.6%, 87.2%, and 84.6%, respectively, *p* < 0.05). The additional finding was that SDF requires much less chair time than ART and UCT (*p* < 0.01) [14].

Another study evaluated the differences between the application of 38% SDF and 38% SDF followed by 5% NaF (at baseline and after 6 months). The authors summarized that 38% SDF with 5% NaF had a higher caries arrest rate than 38% SDF alone (77.7% and 73.2%, respectively, *p* = 0.035) [15].

One publication analyzed the effectiveness of 38% SDF applied in different intervals. This study concluded that the shorter the intervals between the applications are, the higher the carries arrest rates that will occur [16].

The details of randomized controlled trials are presented in Table 2.

The review article compared the strategies of the prevention and treatment of caries in early childhood (17 articles included in the review). It analyzed only minimally invasive methods. Results significant for our publication were those which concerned NaF, NSF, and SDF. The authors concluded that all these methods had demonstrated effectiveness in stopping the progression of ECC, they are generally well-received by both patients and their parents, and SDF’s most frequent adverse effect is the permanent black staining of treated tooth surfaces [17].

### 3.3. Parental and Child Perspectives on SDF Therapy and the Role of Motivational Interviewing in Managing Early Childhood Caries

Four publications were identified within this field. Two of them addressed parental perspectives on the use of SDF, one addressed the dental fear and anxiety of children before and after the SDF therapy, and one study focused on the role of motivational interviewing (MI) in the treatment process of ECC.

The first article was a qualitative study and involved parents of kindergarten children who received outreach dental care. Five children were purposively selected per kindergarten—four received screening and SDF therapy with parents’ consent, one received screening only. Teachers organized parent recruitment and focus group discussions. Researchers developed a guide covering two themes: parental experiences and opinions about the service. Parents were generally satisfied with the outreach dental service, especially because it offered free dental check-ups at their children’s kindergartens. Many felt that the program enhanced their children’s oral health awareness and hygiene practices, such as tooth brushing. Acceptance of SDF therapy was primarily due to its cost-free nature and proven effectiveness in arresting dental caries. However, some parents were worried about the black stains left on treated teeth after SDF therapy (especially anterior teeth), which, in their opinion, made caries appear more severe. A few parents were also concerned about the safety of SDF and did not want their children being exposed to silver or fluoride. Conversely, other parents accepted the staining as a minor issue, especially on posterior teeth, and prioritized oral health over aesthetics. They also noted that primary teeth would exfoliate, reducing long-term cosmetic concerns. Overall, the study concluded that the use of SDF in the kindergarten setting is a generally acceptable approach from the parents’ perspective [18].

The second publication was a cross-sectional study, in which parents provided demographic details, information about their children’s dental history, and their own levels of anxiety. After watching a video explaining the effectiveness and application of SDF, they completed a survey evaluating their acceptance of the treatment and any related concerns. The findings showed that most parents were willing to accept SDF treatment for their children. Acceptance increased significantly with higher levels of parental anxiety. However, 85% of parents expressed concern about the black staining caused by SDF, and those with stronger concerns were more likely to reject the treatment. Specifically, 70% of parents who were extremely worried about staining would not choose SDF therapy for their child. Interestingly, parents with high or extreme dental anxiety were much more likely to approve SDF treatment compared to those with low or no anxiety. The study concluded that SDF treatment is largely accepted by parents of young children, with acceptance influenced by factors such as the parents’ age, their level of dental anxiety, and worries about staining [4].

The third article, which was a cross-sectional study, concerned dental fear and anxiety (DFA) among children, comparing the state before and after the SDF therapy. A dentist conducted the clinical examination and administered SDF therapy to the carious lesions. The children’s dental fear and anxiety (DFA) levels were measured prior to the treatment and immediately after, using a self-reported facial image scale (FIS—showed five faces, from very happy to very distressed). The results revealed that most children (87%) before the therapy had no or low DFA. Following the FIS assessment, children reported that they were very happy (65%) or happy (14%) after the SDF treatment, 7% were neutral towards the treatment, 3% felt distressed, and 11% felt very distressed. The study concluded that children with ECC typically showed minimal or no DFA following SDF treatment conducted at school, regardless of their demographic background or initial DFA levels prior to the therapy [19].

The last publication focuses on the influence of motivational interviewing (MI) on the parental risk-related behaviors and their knowledge of ECC. It was a systematic review (seven studies analyzed). The results of this study support the use of MI to improve the “dental attendance for fluoride use” (two studies reported in this publication found that the number of visits for fluoridation was significantly higher in the MI group) and the participants’ knowledge (two studies included in this review indicated the significant improvement in this field after conducting MI, with *p* = 0.031 and *p* < 0.001, respectively). The results for other behaviors (e.g., tooth brushing) were inconclusive [20].

A summary of the findings from these four publications is presented in Table 3.

To summarize, the charting results identified 15 studies, including randomized controlled trials, systematic reviews, reviews, cross-sectional studies, and a qualitative study. Most trials compared the application of 38% SDF with 5% NaF; only one study compared 38% SDF with NSF. The smallest sample size was reported in the clinical trial conducted in Canada (84), whereas the largest was observed in the study from China (“1070” participants). Follow-ups most commonly occurred at 6 and 12 months. The earliest outcomes were assessed at 2 months, while the longest follow-up period extended to 30 months. Only one publication focused on motivational interviewing in the context of early childhood caries.

## 4. Discussion

Early childhood caries is a common health issue all around the world. Due to the fact that it concerns young children, treatment methods ought to be simple, quick, and non-stress generating. Non-invasive dentistry addresses these needs. In 2021, the World Health Organization (WHO) included silver diamine fluoride (SDF) on its Model List of Essential Medicines for Children as a safe treatment for dental caries. The organization emphasized that untreated dental caries is a global public health issue, and increasing access to SDF could help mitigate this problem.

Contemporary dental practice emphasizes non-operative strategies designed to reverse, arrest, or slow the progression of carious lesions. This approach is supported by continuous advances in dental technology, holistic preventive measures, and deeper insights into the biological mechanisms of caries development [21].

Despite the wide range of currently available non-invasive treatment methods for ECC, SDF remains the very popular option, especially for moderate and advanced lesions. It can be applied regardless of access to advanced equipment, dental clinic conditions, or highly qualified personnel and it is cost-effective [22]. In response to patients’ needs, researchers are working on modifications to SDF to minimize or completely eliminate its most noticeable side effect—the black staining of carious lesions. One of the results of these efforts is nano-silver fluoride (NSF) which combines the properties of fluoride and silver without discoloring the teeth, while maintaining a similar effectiveness in treating caries to SDF. NSF is not yet a popular treatment option, but a few studies have documented its similar antibacterial efficacy to SDF [17]. In clinical trials, it seems to be even more effective in arresting carious lesions than SDF and induces greater parental satisfaction in their children’s appearance [11].

Researchers generally agree that SDF is effective in treating ECC. However, randomized controlled trials are conducted at various intervals, under different configurations, often comparing SDF with other treatment methods. Additionally, they often differ in terms of study setting, the surfaces of the examined teeth, and the severity of the lesions being analyzed. For these reasons, making straightforward comparisons is very challenging and often impractical.

Analysis of clinical trials indicates that the application of 38% SDF covered by 5% NaF at shorter intervals is more effective in arresting carious lesions in ECC (one-month and four-month intervals were more effective than six-month intervals in arresting carious lesions) [16]. Similar conclusions, but referring to the application of 38% SDF alone, were made by the authors of another publication—multiple applications per year yield better results than a single annual application [23].

Some studies have examined the effectiveness of SDF combined with the following application of 5% NaF versus SDF alone. The greater efficacy of SDF covered with NaF compared to SDF alone may be explained by the delayed rinsing effect, allowing SDF to remain longer on the treated surface (because it is covered with NaF varnish). This approach also helps minimize one of SDF’s side effects—its metallic taste [15].

Other authors analyzed 38% SDF in comparison with 5% sodium fluoride (5% NaF) in their clinical trials. In one of them, agents were applied only once and combined with motivational interviewing (MI) intervention [12]; in a second study, agents were applied semiannually over 18 months [9]; in the third study, there was also a single application and the observation period was 12 months (in this study, agents were allocated only on six upper anterior teeth) [10]. In two of these studies, the authors concluded that there was no significant difference between the application of 38% SDF and 5% NaF in arresting caries on the examined surfaces [9,10]. However, it was emphasized that the research findings clearly indicate better treatment outcomes in non-cavitated compared to cavitated carious lesions [9]. Additionally, the location of the carious lesions plays a significant role in treatment success, with enamel caries on anterior teeth or on the buccal or lingual surface of posterior teeth showing more favorable results. This may be due to the fact that these areas are more easily accessible for cleaning and are regularly exposed to saliva and fluoride, which enhances the remineralization process [9]. In the study with parental motivational interviewing (MI), the researchers also concluded that 5%NaF/MI can be an alternative to 38% SDF in arresting ECC lesions, but they highlighted that in advanced lesions the arrest rate was significantly higher in the SDF than in the NaF/MI group [12]. However, in moderate lesions, the authors attribute the similar effectiveness of these two agents to motivational interviewing (MI), which induced behavior change and improved oral hygiene [12].

Although parental motivational interviewing has not yet been thoroughly studied, the research indicates that it could be a helpful tool in supporting the process of ECC treatment [20]. However, additional strategies that enhance parental awareness are worth considering, beginning as early as the prenatal stage—for instance, by educating expectant mothers, who play a crucial role in developing their children’s health-related habits [24].

The next trial investigated whether there is a difference in the effectiveness between the application of 25% silver nitrate (25% AgNO_3_) followed by 5% NaF and the use of 38% SDF alone [13]. It demonstrated that the semiannual application of silver nitrate along with sodium fluoride is at least as effective as the semiannual application of 38% SDF alone in arresting early childhood caries (ECC). This is likely due to the fact that both treatment combinations contain compounds of fluoride and silver, which may exhibit similar therapeutic properties.

One clinical trial included in our review compared the application of 38% SDF with atraumatic restorative treatment (ART) and with ultraconservative treatment (UCT) [14]. This publication can be compared with the study that made the comparison between the application of 38% SDF with placebo and with 5% NaF [25]. Placebo and UCT may be comparable in these trials, if we assume that the placebo group children brushed their teeth with fluoride toothpaste twice daily. The first one revealed that UCT had a significantly lower caries arrest rate than 38% SDF and ART. However, the second one showed no significant differences between three groups. These outcomes may be attributed to several factors. Firstly, the procedures were conducted on different surfaces (SDF/ART/UCT only on the occlusal surface of primary molars, SDF/placebo/NaF on many surfaces, both molars and canines). Secondly, different stages of caries were assessed—in the placebo trial, participants also presented initial caries, whereas in the study involving ART, only advanced lesions were evaluated.

This scoping review identified that the majority of the included studies were clinical trials, with a predominant focus on silver diamine fluoride (SDF). Most of these trials compared SDF with other non-invasive methods. The mapping of the range of non-invasive approaches underscores the need to broaden investigations into alternative non-invasive strategies. Although the evidence on non-invasive methods for the secondary and tertiary prevention of early childhood caries is increasing, important gaps in the literature remain. Many studies are limited by small sample sizes, short follow-up periods, and heterogeneous interventions and outcome measures, which hinder comparability. There is also a noticeable scarcity of studies addressing motivational interviewing in the context of early childhood caries (ECC). Future research should therefore employ standardized procedures, reporting, and larger populations to strengthen the evidence base.

## 5. Conclusions

In conclusion, this scoping review offers a novel perspective by jointly considering SDF, NaF, NSF, and motivational interviewing in the context of the secondary and tertiary prevention of ECC. This integrated approach not only facilitates comparison across interventions but also reveals research gaps.

The scoping review of the literature on non-restorative secondary and tertiary prevention of ECC highlighted several important issues:

- The vast majority of publications identified in this review focus on SDF treatment, indicating significant interest in this topic in recent years.
- There is a clear need for studies conducted under more standardized conditions—both in terms of the study population (e.g., the severity of lesions, treated teeth surfaces) and the treatment methods being compared, including the intervals between applications of the agents and the timing of final outcome assessments. Such consistency would enable more accurate comparisons between treatment approaches.
- Given the limited evidence on motivational interviewing in young children, parental involvement appears to be a promising direction for both research and practice.

Nonetheless, based on the available literature, some clinically relevant conclusions can be drawn, providing guidance for dental practice:

- Treatment of ECC with 38% SDF is considered to be safe, quick, and simple, making it a child-friendly and widely available option.
- Shorter intervals between applications of 38% SDF enhance its effectiveness in arresting ECC.
- Applying 38% SDF in combination with 5% NaF varnish improves both the efficacy of the treatment and patient comfort (minimized metallic taste). However, such practice extends the duration of this procedure.
- Nano-silver fluoride (NSF) is a promising alternative that may eliminate the most significant side effect of SDF—the black staining of carious lesions. Still, further research is needed in this area.

Modern dentistry focuses on maintaining oral health and preserving healthy, natural teeth for as long as possible. The later the surgical intervention in the tooth tissue occurs, the greater the chance is of preserving one’s own natural dentition for longer. That is why it is so important to seek solutions that are positively received by young patients, as it helps to foster a positive attitude toward oral hygiene and dental treatment later in life.

## Figures and Tables

**Table 1:** Inclusion and exclusion criteria.

Inclusion Criteria	Exclusion Criteria
Children under 6 years of age with existing carious lesions	Children aged 6 years and older
Studies employing non-invasive/non-restorative interventions with simple armamentarium	Studies employing surgical/invasive interventions requiring advanced equipment
Publications addressing early childhood caries	Publications which do not address early childhood caries
Publications reporting data on effectiveness	Studies without data on intervention effectiveness
Publications published within the past 5 years	Publications published more than 5 years ago
Studies published in English	Studies published in languages other than English
Open access publications	Publications not available in open access

**Table 2:** Summary of published randomized controlled trials.

Study	First Author	Publication Year	Country	Type of Study	Sample Size	Procedure	Intervention	Key Findings	Conclusions
[16]	Schroth RJ	2024	Canada	Randomized Clinical Trial	84 children	38% SDF along with 5% NaF applied at different intervals	Group 1—visits one month apart Group 2—visits four months apart Group 3—visits 6 months apart	At the final visit, 98% of lesions were arrested in the one-month group, 95.8% in the four-month group, and 72% in the six-month group.	Two applications of 38% SDF and 5% NaF, administered at one-month and four-month intervals, demonstrated similarly high effectiveness in arresting dental caries. Treatments conducted 6 months apart showed reduced efficacy.
[11]	Quritum M	2024	Egypt	Randomized Clinical Trial	360 children	NSF vs. 38% SDF	Group 1—single application of NSF at baseline Group 2—two applications of 38% SDF (at baseline and after 6 months)	1. The caries arrest rate was significantly higher in the NSF than SDF group. 2. The NSF group showed greater parental satisfaction than SDF group (due to the lack of black staining of teeth).	NSF was more effective than SDF in arresting caries in preschool children, without black staining and with higher parental satisfaction, making it a viable alternative.
[14]	Hamza BE	2024	Egypt	Randomized Clinical Trial	135 children	ART vs. 38% SDF vs. UCT (only occlusal surfaces in primary molars)	Group 1—ART (control group) Group 2—38% SDF Group 3—UCT	1. After a year, the overall success rates of UCT, SDF, and ART were 61.6%, 87.2%, and 84.6%, respectively (*p* < 0.05). 2. Anxiety levels were lower in SDF than ART group (*p* = 0.003).	1. After 3 months, SDF, ART, and UCT had similar caries arrest rates. 2. After 1 year, the arrest rate of UCT was significantly lower. 3. SDF required much less chair time than ART and UCT. 4. The anxiety of children receiving SDF and UCT was significantly reduced compared to those receiving ART.
[12]	Yassin R	2023	Egypt	Randomized Clinical Trial	165 children	38% SDF vs. 5% NaF/MI (single application)	Group 1—38% SDF Group 2—5% NaF combined with two motivational interviewing (MI) sessions (one at a baseline and second after three months)	After 6 months, in advanced lesions the caries arrest rate was higher in the SDF than NaF/MI group (60.3% and 50.0%, respectively, *p* = 0.01)	5% NaF/MI can be an alternative to 38% SDF in arresting advanced and moderate ECC lesions without staining with stronger effect on moderate lesions (ICDAS 3/4).
[15]	Abdellatif EB	2023	Egypt	Randomized Field Trial	220 children	38% SDF and 5% NaF vs. 38% SDF (at baseline and after 6 months), 5% NaF was additionally applied on all teeth in oral cavity, and it was also applied after three months	Group 1—38% SDF with 5% NaF Group 2—38% SDF alone	Caries arrest was achieved in 77.7% of lesions with SDF + NaF and 73.2% with SDF alone (*p* = 0.035).	38% SDF with NaF had a higher caries arrest rate than SDF and this difference was significant in moderate but not in advanced lesions.
[9]	Phonghanyudh A	2022	Thailand	Randomized Clinical Trial	290 children	38% SDF vs. 5% NaF (agents were applied semiannually over 18 months)	Group 1—38% SDF Group 2—5% NaF	Caries arrest rates at the tooth surface level in Group 1 and Group 2 were 59.1% and 58.8%, respectively (*p* = 0.873), at 18 months.	1. There was no statistically significant difference in arresting enamel caries of primary teeth between the use of 38% SDF and 5% NaF applied semiannually in preschool children. 2. The use of 38% SDF had no notable adverse effects and no impact on parental satisfaction with dental appearance of children.
[10]	Zheng FM	2022	China	Randomized Clinical Trial	434 children	38% SDF vs. 5% NaF (the agents were allocated on 6 upper anterior teeth)	Group 1—38% SDF Group 2—5% NaF	1. After one year, caries increment did not differ significantly between groups (SDF: 26.8%, NaF: 24.9%; overall 38%; *p* = 0.65). Parental satisfaction was similar as well, with 71% for SDF and 69% for NaF (*p* = 0.29).	1. 12-month observation found that a single application of 38% SDF was not superior to 5% NaF for caries prevention in primary upper anterior teeth. 2. Child’s cooperation and parents’ satisfaction were similarly high in SDF and NaF therapy.
[13]	Gao SS	2020	China	Randomized Clinical Trial	1070 children	25% AgNO_3_ and 5% NaF vs. 38% SDF	Group 1—25% AgNO_3_ and 5% NaF Group 2—38% SDF	The mean arrested ds in Groups A and B were 3.7 ± 3.6 and 3.6 ± 3.7, respectively (*p* = 0.694).	Semiannual application of 25% AgNO_3_ followed by 5% NaF is at least as effective as semiannual application of 38% SDF in arresting ECC.

Abbreviations: NaF—sodium fluoride, SDF—silver diamine fluoride, NSF—nano-silver fluoride, AgNO_3_—silver nitrate, UCT—ultraconservative treatment (in this study—only cleaning the teeth with fluoride toothpaste twice daily); ART—atraumatic restorative treatment, ds—decayed surfaces.

**Table 3:** Summary of the publications concerning parental acceptance, dental fear, and motivational interviewing of silver diamine therapy (SDF) in early childhood caries.

Study	First Author	Publication Year	Country	Type of Study	Sample Size	Intervention	Key Findings	Authors’ Conclusions
[18]	Chai HH	2022	China	Qualitative Study	49 parents	A study was conducted in collaboration with a community outreach program that provided silver diamine fluoride (SDF) treatment to kindergarten children and oral health education to their parents. Using purposive sampling, five parents from each Hong Kong kindergarten were invited to focus group discussions. Data were manually coded and analyzed thematically.	Parents valued the oral health education for improving knowledge, awareness, and parent-assisted brushing. Some declined SDF due to black staining and safety concerns, while others prioritized decay control over aesthetics, noting that primary teeth are temporary.	Implementing outreach dental programs using SDF for managing ECC in kindergarten settings is generally perceived as an acceptable approach from parents’ perspective.
[4]	Ladparkdy S	2024	Laos	Cross-Sectional Study	324 parents	Participants reported demographics, children’s dental history, and anxiety levels. Parents completed a 5-point Likert survey on SDF acceptance after a video demonstration, and dental anxiety was assessed with the modified scale.	1. Most parents (84.9%) reported some level of dental anxiety. 2. Acceptance of SDF increased with rising anxiety levels and age. 3. Overall, 80% of parents accepted SDF for their children, though concern about staining reduced acceptance.	1. Parents of preschool children largely viewed SDF treatment as acceptable. 2. Older parents showed less concern about staining, and those with greater dental anxiety were more inclined to accept SDF.
[19]	Sun IG	2023	China	Cross-Sectional Study	340 children	A dentist examined the children and applied SDF to carious lesions. The children’s DFA was self-rated with the FIS scale before and after treatment.	The majority of preschool children with ECC demonstrated minimal or no DFA after SDF treatment conducted in a school-based setting.	Based on the child-reported DFA assessment, preschool children with ECC exhibited no or low DFA after SDF therapy in a school-based setting, regardless of their demographic background, dental visits, and their DFA before SDF therapy.
[20]	Mortazavi S	2021	Iran	Systematic Review	-	-	Of 329 articles, 7 met the inclusion criteria: 4 on tooth brushing, 4 on cariogenic feeding, 1 on checking teeth for pre-cavities, and 2 on dental attendance for varnish fluoride use and oral health-related knowledge.	Results indicate that MI is effective in improving the “dental attendance behavior for fluoride use” and participants’ knowledge, but for other behaviors (e.g., tooth brushing) the results were inconclusive.

Abbreviations: SDF—silver diamine fluoride, ECC—early childhood caries, DFA—dental fear and anxiety, MI—motivational interviewing.

## Data Availability

No new data were created or analyzed in this study.

## Supplementary Materials

The following supporting information can be downloaded at https://www.mdpi.com/article/10.3390/healthcare14010064/s1. Table S1: Characteristics of Included Studies.

## Abbreviations

The following abbreviations are used in this manuscript:

ECC	Early childhood caries
SDF	Silver diamine fluoride
NSF	Nano-silver fluoride
NaF	Sodium fluoride
AgNO_3_	Silver nitrate
MI	Motivational interviewing
ART	Atraumatic restorative treatment
UCT	Ultraconservative treatment
DFA	Dental fear and anxiety
